# Current and emerging global themes in the bioethics of regenerative medicine: the tangled web of stem cell translation

**DOI:** 10.2217/rme-2017-0065

**Published:** 2017-11-09

**Authors:** Sarah Chan

**Affiliations:** 1 Usher Institute for Population Health Sciences & Informatics, University of Edinburgh, Teviot Place, Edinburgh, EH8 9AG, UK

**Keywords:** ethics, regenerative medicine, regulation, research translation, stem cells

## Abstract

Probably the most serious problem facing the field of regenerative medicine today is the challenge of effective translation and development of viable stem cell-based therapies. Particular concerns have been raised over the growing market in unproven cell therapies. In this article, I explore recent developments in the stem cell therapy landscape and argue that while the sale of unproven therapies undoubtedly poses ethical concerns, it must be understood as part of a larger problem at the interface between biomedicine, healthcare, publics, policy and the market. Addressing this will require a broader perspective incorporating the shifting relationships between different stakeholder groups, the global politics of research and innovation, and the evolving role of publics and patients with respect to science.

The field of regenerative medicine is perhaps one of the most topical areas of contemporary bioscience. Recent developments in regenerative medicine research touch on a wide range of ethical issues. First, the use of human embryos in deriving human embryonic stem cells remains the subject of strong debate. While some see the advent of induced pluripotent stem cells as alleviating these concerns, these issues nevertheless remain bound up with the field of regenerative medicine as a whole. Research and treatments using human embryonic stem cells continue to be developed and influence the direction of regenerative medicine research and translation. Moreover, induced pluripotent stem cells themselves do not necessarily solve the ethical problems associated with the use of embryos. This is perhaps even more so now that the possibility of generating embryo-like structures by patterning pluripotent stem cell has been raised [1–6].

Another area of ethical interest concerns developments in organogenesis and tissue engineering. Among the most intriguing are the prospects for xeno-organogenesis, using a combination of gene editing and stem cell science to create interspecies chimeric animals with humanized organs as a source of transplants [7–12]. Human–animal chimeras have been the subject of considerable ethical discussion [13–19]; the fact that this work involves the use of human pluripotent stem cells in animal embryos at an early stage raises several of the concerns highlighted as most salient, notably that human pluripotent cells might contribute to brain or reproductive tissues.

Probably the biggest ethical challenge in the field of regenerative medicine at present, however, is the emergence of a thriving market in unproven ‘stem cell’ treatments. The worldwide proliferation of clinics offering treatments claiming to be based on stem cells, often in the absence of rigorous or in some cases any scientific evidence, has been widely criticized by scientists and ethicists alike [20–28]. Critics have rightly raised concerns over patient safety, as well as the potential negative impact on the development of genuine therapies, if the majority of treatments being sold are unsafe or useless, leading to loss of faith from public and investors. Often, it is perceived that the purveyors of such treatments are doing nothing more than exploiting the vulnerable and depriving gullible and desperate patients of their money.

Yet recognizing this as a problem does not necessarily imply that the solution must be to shut all such clinics down, or that the ideal situation is one in which no ‘unproven treatments’ are ever made available. Characterizing all patients as powerless and uninformed pawns being taken advantage of by unscrupulous operators does not take account of the complex lived realities that patients experience or how they negotiate the diverse factors that drive them to seek treatment in particular ways [29–33]. Nor is it simply the case that all unproven therapies are ‘quack’ treatments, or that there is one single ‘right’ pathway toward effective regenerative medicine therapies and anything that deviates from this must be ‘wrong’. The complexities of global science developing in diverse local contexts, with different regulatory systems and cultural norms, in an international market driven by patient demand and shaped by the vast commercial potential that demand creates, mean that such a black-and-white approach is unjustified. Instead, we need to attempt to understand why and how this situation has emerged in order to determine the best ethical approaches to the problem and the possible solutions.

## Defining the problem

The first hurdle in addressing the issue is the concept of ‘unproven treatments’ itself and the potential breadth of definition it encompasses. Regenerative medicine is a developing field, with many promising avenues for clinical translation still under investigation and new possibilities constantly emerging. As such, describing a treatment as ‘unproven’ might indicate any point on a spectrum ranging from “*legitimate in-process research activities*” [34] toward the development of verified therapies, to complete ‘snake oil’ treatments that lack any scientific basis. Within this spectrum, there is a broad terrain in which genuine scientific uncertainty, evolving understandings of stem cell science and differing ethical and regulatory norms are co-negotiated, and across which there is no single agreed point at which offering treatment becomes ‘valid’ [35].

The term ‘unproven’ thus encompasses a large gray zone: treatments cannot simply be classed as ‘rogue’ or ‘legitimate’. Clearly, we cannot say unproven treatments should never be offered, otherwise no new treatments would ever be developed. At the same time, it seems *prima facie* undesirable that patients should invest money and hope in ineffective treatments on the basis of false claims or expectations, while recurring reports of adverse incidents after the administration of cell-based treatments demonstrate that safety remains a real concern [36–38]. There is also the question of whether the treatments on offer are being accurately represented by providers and accurately perceived by patients. Whether people should have the freedom to gamble on treatments they know to be uncertain is one issue, but if they are choosing to receive treatments based on misinformation or misunderstandings about the nature of the procedure, whether or not it is established as an effective treatment and the likely chances of success, that is a different matter.

In the face of this ambiguity over what ‘unproven’ might imply, taking steps toward defining which forms of unproven cell therapies pose most concern may provide something of a path to navigate this uncertain terrain [25,34]. Given the emerging dynamic of patient-driven demand for wider access to treatments, as will be discussed further, this process requires not just the cooperation of the scientific community but the active involvement of other stakeholders. While professional scientific bodies may wield authority in the domain of science, the provision of cell therapies takes place in a wider arena. Patients and providers are not constrained to operating only within science; their exercise of agency extends to other spheres, notably including technology and health markets, and they may refuse to yield to scientists’ attempts to impose extraterritorial jurisdiction. In other words, the problem posed by unproven cell therapies is one that extends beyond the boundaries of science and cannot be solved only by standard-setting on the part of the scientific community.

## From ‘stem cell tourism’ to trouble at home

The phenomenon of widespread marketing and provision of unproven cell therapies has been recognized for some time; since that point, the landscape has continued to evolve, bringing novel dimensions to the issue.

One such development concerns where treatments are being provided. In the early days of the stem cell boom, ‘stem cell tourism’, in which clinics would set up their operations in countries with more permissive regulatory environments and patients would travel to access them, was rife [29,39–42]. This phenomenon was a well-characterized aspect of the field, to the extent that the term ‘stem cell tourism’ has become almost synonymous with unproven cell therapies.

It is now recognized, however, that the problem is not only one of individuals engaging in medical tourism and crossing borders to receive treatment. Domestic markets for unproven cell therapies are thriving: in the USA and Australia, for example, there are numerous clinics offering treatments supposedly based on stem cells [37,43]. Meanwhile, although countries such as China and Mexico have been characterized as hubs for stem cell tourism from abroad [41,44], their clinics also service local patients.

An implication of this is that we cannot export responsibility for the problem by attributing the continued rise of unproven treatments to inadequate regulation in other countries, or on a lack of transnational harmonization that renders the market more susceptible to medical tourism. Attempts to control the provision of stem cell-based treatments, however, reveal a deep seam of disagreement within national borders as to how and even whether such treatments should be regulated: the 's proposal to tighten regulations for cell therapies attracted vociferous objection from patients who saw it as blocking access to treatments, even as scientists urged caution over safety and lack of evidence [45,46].

Indeed, there is a powerful current of opposition in the USA from patients and providers seeking to further weaken, rather than increase, regulatory control over stem cell treatments. Initiatives such as the REGROW bill proposed in 2016 (though ultimately unsuccessful) and the ‘’ movement [47] seek to circumvent the established FDA pathways for testing and approval of new treatments. Patient support for these comes largely from the desire for access to treatments, even where those treatments may be at an early stage of experimentation and their actual effectiveness not yet demonstrated. For clinics, meanwhile, deregulation would mean less oversight, less stringent evidence requirements and less time required before being able to market their products. Presumably, some of the stem cell providers leading this drive believe that their treatments are effective, even if rigorous evidence is yet to be produced, and are motivated by the desire to help patients as soon as possible. Given the commercial nature of many of these enterprises, however, the lowered costs and reduced barriers to being able to market their products are undeniably also in providers’ business interests.

Against this pressure from patient groups and providers, scientists who warn of the dangers of deregulation and express doubts about the way in which unproven treatments are being provided may find themselves cast in perhaps unexpected opposition to the patients whose interests they are seeking to protect [29,32]. Amidst this increasingly polarized debate, the current climate of regenerative medicine research and treatment thus sees new tensions and alliances emerging: rather than between different countries with differing standards of regulation, the arena in which provision of stem cell treatments are now being contested lies between different stakeholder groups with differing interests. How these relationships are negotiated will be crucial to determining the course of the regenerative medicine field over the next few years.

## Tapping the multinational research market

Another aspect of regenerative medicine emerging in the international context is the possibility of cross-border research marketing to take advantage of more permissive regulation. The potential for scientific tourism in the field of stem cell science is well recognized, and there have been documented instances of clinics relocating operations to less-regulated jurisdictions in order to avoid capture [48]. As countries adopt different positions on the regulation of translational pathways toward regenerative medicine, another form of cross-border activity is companies taking advantage not of lacunae in regulation and oversight, but of explicit regulatory mechanisms in some countries that allow for the provision and marketing of stem cell-based treatments at an early stage of research.

An example of this is Japan, which recently introduced a new regulatory scheme for regenerative medicines that includes provisions for conditional approval of treatments at an early stage of translation. Under the changes brought about by the Regenerative Medicine Promotion Law in 2013 [49,50], regenerative medicine products can be brought to market at a much earlier stage, after only small clinical studies but without the need to demonstrate efficacy and long-term safety via the usual clinical trial process. Full safety and efficacy data will then, it is proposed, be collected on a postmarket basis to validate conditionally approved treatments. According to its proponents, the strategy is designed to encourage investment in regenerative therapies and accelerate the process of development by expediting commercialization without the requirement for lengthy and costly clinical trials. Critics, however, have expressed doubts as to whether this will work, or whether it will result in Japan being “*flooded with unsuccessful treatments*” [51] at the expense of its patients and healthcare system [52]. See Table 1 for a comparison of examples of regenerative medicine regulation in Japan, the UK and the USA.

**Table 1.** **Access to new regenerative medicine/stem cell treatments: key regulations and access pathways in the USA, UK and Japan.**

**Country**	**Regulatory bodies**	**Key regulations**	**Scope**	**Effect**
USA	US FDA	Code of Federal Regulations (21 CFR 1271) PHSA 1944, s351 and 361	HCTPs	HCTPs that are ‘minimally manipulated’; ‘intended for homologous use only’ and not combined with ‘another article’ are regulated under s361 of the PHSA (21 CFR 1271.10). They are considered part of the practice of medicine and out with the FDA's jurisdiction. HCTPs that do not meet these criteria are more strictly regulated, under s351 of the PHSA, as biological drugs requiring premarket evaluation. There is an exception to this for HCTPs that are removed and reimplanted into the same patient during the ‘same surgical procedure’ (21 CFR 1271.15)

		Draft guidance issued 2014–2015 (same surgical procedure exception; minimal manipulation; adipose tissue-derived cells; homologous use)	–	In an attempt to clarify the regulation of HCTPs and whether premarket FDA approval is required, the FDA published draft guidance on these provisions and sought comment on these, including a series of public hearings, in September 2016. Many of the clinics currently providing cell-based treatments rely on the ‘same surgical procedure’ exception and the definition of ‘minimal manipulation’ to avoid the FDA having jurisdiction over their activities

		Early access regulations [55]	Products regulated by the FDA	There are various pathways established by regulation for allowing early access to treatments regulated by the FDA, notably the accelerated access pathway for treatments that are not fully validated but are thought likely to provide a significant benefit. Approvals issued in this pathway are intended to be subject to postmarket evaluation of efficacy

		21st Century Cures Act 2017, s3033	Defines a class of ‘RMATs’	RMATs are “*defined as a cell therapy, therapeutic tissue engineering product, human cell and tissue product, or any combination product using such therapies or products*”, aimed at ‘serious or life-threatening’ diseases or conditions, with “*the potential to address unmet medical needs*”. Products falling under s361 of the PHSA are excepted. Designated RMATs may be eligible for expedited review. In order to obtain a designation, an IND application is needed, in turn requiring an evidence base of scientific data

UK	EMA MHRA	Tissue Framework Directive (2004/23/EC) Advanced Therapy Medicinal Products Regulation (EC No 1394/2007) Advanced Therapy Medicinal Products Directive (2001/83/EC)	*‘Advanced therapy medicinal products’*: gene therapy; somatic cell therapy; engineered tissues	Current UK regulation of cell-based therapies includes compliance with the EC regulatory framework, which requires approval from the EMA based on review of safety and efficacy data. The MHRA is the UK regulatory body for medicinal products including ATMPs, and applies the standards and procedures established by the EC framework and the EMA. Under the ATMP specifications, ‘somatic cell therapy’ refers to products that are ‘substantially manipulated’, meaning that minimally manipulated products are not covered by ATMP regulations; the Tissue Framework Directive also has an exemption for autologous uses as part of the same surgical procedure [56]

		Early access regulations [55,57]	–	The EMA regulatory framework also includes accelerated access pathways, including a 1-year conditional marketing authorization for products that address unmet medical need in relation to ‘seriously debilitating or life-threatening conditions’

Japan	MHLW	Regenerative Medicine Promotion Law (2013)	Regenerative medicine therapies	Establishes “*the responsibility of government to provide people with opportunities to receive treatment with regenerative medicines based on the latest scientific knowledge. In addition, the law requires the government to develop basic policy regarding promotion of timely and safe research and to establish suitable legislative, budget, and taxation measures for this purpose*” [49]. The ASRM and new PMDA were enacted in consequence of this

		Act on the Safety of Regenerative Medicine 2013	Regenerative medicine	The ASRM applies to both clinical trials and medically innovative uses of regenerative therapies, and applies to technologies aimed at “*reconstruction, repair or formation of structures or functions of the human body*” [49], or cell-based therapies. It classifies these therapies into three levels of risk, and specifies the procedure to be followed in each. Lower-risk treatments (Classes II and III) require the treatment protocol to be submitted to the MHLW without further evaluation; in the case of Class I treatments, the highest risk category, the MHLW must also approve or require changes to the proposed plan

		Pharmaceuticals, Medical Devices and Other Therapeutic Products Act 2013	Regenerative medicine products (new class defined by PMDA)	The most salient effect of the PMDA with respect to stem cell therapies is to establish the conditional approval pathway, by which regenerative medical products may obtain marketing authorization after early clinical studies that establish some degree of safety and a likelihood of efficacy, rather than after full clinical trials. The authorization is valid for 7 years, during which time postmarket evaluation of safety and efficacy is expected

ASRM: Act on the Safety of Regenerative Medicine; ATMP: Advanced Therapy Medicinal Products; EC: European Commission; HCTP: Human cell or tissue-based product; IND: Investigational new drug; MHLW: Ministry of Health, Labour and Welfare; MHRA: Medicines and healthcare products regulatory agency; PHSA: Public Health Services Act; PMDA: Pharmaceuticals, Medical Devices and other Therapeutic Products Act; RMAT: Regenerative medicine advanced therapy.

In the wake of the introduction of this scheme, the UK company Cell Therapy Ltd. (now Cellixir) announced its plans to partner with a Japanese company in order to move into Japan and take advantage of what it described as “*a very innovative and appealing regulatory pathway*” [53]. According to CEO Ajan Reginald, “*(t)he accelerated regulatory pathway for regenerative medicines in Japan*” [54] made expanding into Japan a strategic priority. In effect, the company will gain early access to begin selling its still-experimental products to the Japanese market, with the ability to gain data from this to support the innovation process in the UK and Europe.

While multinational marketing is nothing new, the fact that conditional approval takes place at a stage that is still part of the research process adds complexity to the ethical issues. The ‘offshoring’ of clinical trials is known to have potentially corrosive effects and implications for global justice in research and health [58]. What is being offshored here is not a clinical trial as such, and the movement is not across a global North–South border (though perhaps the East–West divide may be relevant in setting the stage for such a move). Nonetheless, the landscape here shares some similar features, notably the way in which these transactions “*insert themselves into ongoing and unresolved conflicts over market reforms*” [58] and the multiple, tangled pathways of healthcare innovation.

Questions therefore need to be asked about this model of cross-border research translation and its effects. Entry into Japan's conditional approval pathway is based primarily on safety, not necessarily proof of efficacy, which is supposed to come from postmarket data. Hence, while Reginald describes the pathway as providing “*accelerated access for patients to get lifesaving medicines*” [54], it would be more accurate to say potentially lifesaving: these products by definition are not yet fully validated.

Again, some have expressed doubts about the conditional approval system, suggesting that it will increase healthcare costs without providing any increase in benefit by way of new effective treatments [52,59,60]. Evidence from the pharmaceutical sector, where systematic deregulation has been progressing for decades, suggests that permitting early market access for drugs based on the expectation of postmarket evaluation does not accelerate the development of effective treatments, only companies’ ability to make money selling products of unknown efficacy; that it may have detrimental consequences for patient safety; and that promised postmarket evaluations do not always take place, or even when they do and fail to show efficacy, the drug remains on the market regardless [61].

Furthermore, the conditional approval system redistributes the cost of research in a way that strongly favors companies rather than patients. The first product to receive conditional approval in Japan, Terumo's Heartsheet, has now also been approved for government health coverage. The fact that the product can be sold commercially even though it is still essentially at the trial stage means that patients and the national health system “*basically subsidize the company's clinical trial*” [51]. In the case of Cell Therapy Ltd's products, conditional approval may mean that Japanese patients and the Japanese health system bear the costs of research for a UK company that can then realize its profits on the global market.

This form of translational research tourism seems potentially unjust for populations in the destination country who are bearing both the on-ground economic and human costs of the work, and in terms of the dynamic between countries in the international market, if UK science is benefiting at Japan's expense. At the same time, populations in the origin country who are unable to access treatments may feel they are missing out. This leads to pressure to deregulate at home: an explicit reason behind the introduction of the REGROW bill was the concern that too-strict regulations were holding back the development of regenerative medicine in the USA, and that accelerated access provisions and the like were essential to keep pace with countries such as Japan [62].

In contrast to the more egregious examples of clinical trial offshoring, the dynamic of exploiting socioeconomic inequity between nation states is absent in this particular case. We might ask, however, whether there is a different dynamic of inequity to be aware of in the evolving terrain of international regulatory differences, in terms of the politics of science and the flows of power within global scientific discourse. The importance of stem cell science and regenerative medicine to the national agenda in Japan [63] creates pressure toward policies intended to promote rapid advance in this field, in order to gain ground in the arena of global science and the biotechnology market. This drive must be understood in the context of an international scientific competition that has been heavily dominated by certain few and powerful countries, with adverse scientific, political and economic consequences for those countries that lag behind: the push toward expanded provision is a way for Japan itself to keep pace. If, however, other countries are taking advantage of Japan's regulations to advance their own research while the costs are met by Japanese patients and taxpayers, it may be that such a move will increase, rather than combat, global scientific inequities.

Similarly, Sleeboom-Faulkner *et al*. have noted how actors in the field of regenerative medicine in various countries engage in a process they term ‘national home-keeping’, the mechanics of which differ depending on how the country is positioned with respect to the global field in terms of size, resources and capacity; established modes of governance; and scientific ambitions [64]. This form of boundary work, they argue, enables countries to reconcile ‘global’ standards that in reality are set by a scientific community whose norms and values are derived elsewhere, with the local practices and policies necessary for the development of stem cell science and regenerative medicine in their own country.

Negotiating ethical variability thus provides a way for countries with lower resources and less influence in the global politics of science to continue to compete in stem cell innovation. At the same time, however, as in cases of trial offshoring, depending on how this variability is exploited in the global context, it may pose a danger of leaving disadvantaged participant populations with inadequate protection, and contribute to entrenching inequity.

## Patient rights & access to treatment: a new role as health consumers

Another perennial issue for regenerative medicine is the problem of who is able to access treatments and how. Problems of justice in health and biomedicine are widely recognized at local and global levels, in terms of access to healthcare, what gets researched and access to research participation itself. In relation to regenerative medicine, where therapies are still in their infancy, the question becomes: what kind of rights might patients have or assert – moral, legal or other – to access regenerative medicine treatments at early stages of development, and what might such rights entail?

In regenerative medicine, the language of rights is gaining currency especially with the rise of the Right-to-Try movement in relation to stem cells, opening up the question of what rights might apply here and on what they might be based. The concept of a right to access has been framed in various ways. For stem cell therapies, we might consider it in terms of a right to healthcare, a right to experimental treatment or perhaps even a right to participate in research. Each of these will have different justifications and implications requiring ethical and legal evaluation; there is much scope here for further investigation. The way in which the right is framed, though, and the basis on which treatments are being made available, also affects in what mode or role those accessing (or attempting to access) treatments are positioned: are they acting as patients, research participants or in another role?

The research/treatment distinction has been the subject of considerable previous ethical analysis (e.g., [65,66]), together with the resulting ethical tensions between the roles of clinician/scientist and patient/participant. Perhaps most clearly in relation to regenerative medicine, a new role is now evident for those wishing to access health technologies: that of health consumer [31,67]. Understanding how and why this role is emerging and its consequences will be important, if we hope to influence what is happening within the sphere of regenerative medicine and stem cell therapies, proven or otherwise.

We might point to a number of factors which enable the consumer role. Most evidently, there is the increasing provision of treatments on a commercial basis: these products are available to buy. This in turn is facilitated by early market access enabled by regulatory processes such as those discussed above, as well as the presence of treatments that fall outside or escape regulation: for example, the ongoing battle regarding over which forms of cell-based interventions the FDA has jurisdiction, and the selective interpretation of terms in Mexican clinics that allows many treatments to go unregulated [44].

The framing of these treatments as opportunities for commercial research participation, as in pay-to-participate trials, means that treatments can be made available as consumer products even at the unproven stage. Indeed, it may be that consumer value attaches not just to the treatment but to the research participation opportunity itself; more work is needed to understand the motivations of those who pay.

To address the concern that stem cell consumers may be led to pay for ineffective, unsafe treatments out of desperation, scientists are exhorted to ‘sell help not hope’ [26]. What is being sold, though, is only one side of the issue. We need also to consider: what are patients looking to buy? When help does not seem to be available, people will look for hope, creating a potential market; as that market gap becomes filled with opportunistic providers, the balance shifts from ‘management of hope’ [33] to clinics trading in hope. While this may seem like exploitation simply waiting to happen, as Petersen *et al*. note, “*for many patients and carers who have exhausted conventional treatment paths, the hope offered by these providers is apparently preferable to the option of doing nothing*” [33]. The exercise of consumer rights in the regenerative medicine sphere may thus become a way, for those seeking treatments, of negotiating and attaining control when neither patient nor participant roles are sufficiently empowering.

The rise of consumer power as a form of patient agency in relation to stem cell treatments is in turn facilitated by increased connectivity. The Internet and social media are key ways in which patients looking for regenerative medicine treatments interact: the ability to reach out to other patients all around the world, form networks and mobilize is changing the nature of patienthood. Patients can now connect more easily with others, allowing even very small numbers suffering from rare conditions to form group identities, and can ‘shop’ the web for stem cell treatment opportunities around the world and share their experiences with others. They also have the potential for increased access to information about their disease and possible treatment – although ‘stem cell hype’ [68,69], as well as ‘echo chamber’ and confirmation bias effects may influence what information is received and how it is interpreted.

An imminent task for those seeking to shape the direction of regenerative medicine and stem cell therapies, therefore, will be a better characterization of the role of patient–consumers, including the effects of social media in constructing their experience, in order to understand its consequences and how to address them.

We must also address the ethical questions surrounding the mixed consumer–patient role. For example, the principle of informed consent is well established as a tenet of ethical treatment of patients; what level of information are consumers entitled to, and what redress do they have when inadequate information is provided? Do or should patient–consumers receive information about the provenance of the cells they are receiving, the tissue source of these cells and the scientific evidence attached to different cell types in relation to cell-based therapies?

It is well recognized that patients may lack accurate information about the treatments they are seeking and the state of the science. The extent to which lack of information is an issue, based on the perception of patients in various countries, as well as how to address this problem, is something that requires additional empirical investigation. A further question at the interface of ethics and regulation, however, is how to negotiate this issue when access to treatments is mediated both via the role of patients in healthcare, and the role of consumers in the health technology market. What standards are required, and in what regulatory sphere should they be imposed?

## Translational strategies for regenerative medicine?

The ethical issues surrounding regenerative medicine are unfolding in the broader context of global science and translational medicine, in which perspectives on science and its governance and the role of publics, regulators and the market are continuing to evolve. It is increasingly recognized that there is a need for novel and adaptable translational research and health innovation pathways to deal with the exigencies of contemporary bioscience, including new modes of research, global public health, globalized technology markets and shifting paradigms of healthcare, and the interplay between all of these. Regenerative medicine displays particular features with respect to these factors; consideration of the ethical issues entailed by these must inform and be informed by this wider discourse around research translation more generally.

One of the challenges for regenerative medicine and cell-based therapies is that the environment in which these treatments are being developed is disanalogous in some respects to the ‘conventional’ pathway for producing new therapies that has been established in the pharmaceutical context of small-molecule drug development. Certain features especially mean that the standard model of clinical trials as the route to translation may not be the most effective approach for this field [70]. A recent report commissioned by the National Institute of Health Research on the assessment of regenerative medicine products identified a number of key factors which, while not unique to regenerative medicine *per se*, are still likely to influence the course of cell therapy innovation [57].

In the first place, the inherent variability of cell therapies leads to variations in practice and treatment protocols that may make treatments hard to standardize. The fact that the technology is constantly evolving also makes long-term evaluation of treatments difficult [57].

The clinical development of stem cell treatments often involves their use in small, nonrandomized studies aimed at treating patients in need. In such situations, randomization may be ethically problematic. Some of the conditions that regenerative medicine might target are relatively rare diseases, thus low patient numbers may pose the difficulty of recruiting sufficient participants for a clinical trial or the problem of low statistical power when trial populations are small. The situation of *‘unmet medical need*’, where no alternative treatments are available, creates intense demand for new therapies; the market in unproven interventions attracts patients discontented with the slow pace at which treatments progress through the standard pathway, which further diminishes the pool of patients available to be recruited for formal studies.

Moreover, because of the uncertainties involved, industry has been reluctant to invest in stem cell therapies, making it difficult to move from the stage of administering interventions to small numbers of patients in a clinical setting to a larger-scale clinical trial. While scientific organizations such as the International Society for Stem Cell Research generally recommend that provision of stem cell treatments as innovative medical care should be limited and that the aim should be to proceed to clinical trials as soon as possible, the setup of trials requires resources on a different scale. There is thus a potential issue with promising treatments becoming stalled at the stage at which a clinical trial might be appropriate, leaving clinicians and patients in a situation where a potentially effective therapy exists, but no route to administering it outside of the medical innovation context.

All of these factors mean that conventional approaches to health innovation are difficult to apply to the development of stem cell therapies. Effective translation for regenerative medicine is, therefore, likely to require alternative mechanisms. These may include novel ways of assessing the evidence of clinical efficacy, including adaptive research designs that offer alternatives to randomized controlled trials and ways of evaluating evidence from smaller nonrandomized studies, as well as new regulatory pathways to accelerate development and provision [57].

## Should we stop the sale of snake oil?

It may seem that, while more work is needed to optimize translational pathways for regenerative medicine, we should at least attempt to eliminate the provision of treatments that are clearly without any scientific basis. Focusing on this aspect, however, ignores the fact that ‘unproven therapies’, snake oil treatments included, are only one side of the problem – they are a form of ‘supply’ that has evolved in response to the ongoing demand for effective treatments. This demand is most obviously created by unmet medical need; the fact that stem cells are widely perceived as the solution to meet this need, however, is due to the ‘biography’ of stem cell science itself, notably the constitution of stem cells as a sociotechnical object that serves to focus therapeutic hopes as a means of providing impetus to the research field. That these hopes may have outpaced the scientific reality does not negate the strength of the expectations created [71].

Given this, focusing efforts on stopping the ‘snake oil’ market may not address the wider problem: patients’ demands for treatment will still persist. In such a climate, moves to restrict access to unapproved treatments may be perceived as scientists attempting to protect their own interests rather than patient safety, creating a potential rift between patient and scientific communities and setting them in opposition [32].

The ultimate goal of regenerative medicine, from patient and physician perspectives alike, is surely the development of safe, effective, affordable treatments with equitable access, as soon as possible. Seen in the broader context, then, the problem is not just the availability of potentially unsafe and ineffective treatments but how the common goal can be achieved.

We need therefore to ask, not how to stop the supply of bogus and dangerous treatments *per se*, but instead, if all the desired factors (safety, efficacy, affordability, access) are not simultaneously met by the treatments currently on offer, what should be the priorities? Is it better to emphasize safety and efficacy at the expense of timeliness and affordability; or to allow treatments to be sold commercially at an early stage but with fewer assurances of safety and efficacy; or additionally to provide support for treatments even where not fully proven, for the sake of equitable access? Who should determine these priorities and how? And what do we need in terms of ethics, policy and science, in order to move toward the eventual goal?

We might also be able to point to certain behaviors that do not advance this goal in any way and should be unambiguously condemned. One in particular is non-science masquerading as science: many of the clinics providing what they label ‘stem cell therapies’ advertise their services as research and may even register the treatments as a clinical trial, adding apparent legitimacy, but few results seem to be reported.

Even where ‘false advertising’ as science is not explicit, we ought to have low tolerance for clinics that are carrying out unproven treatments without a clear process for collecting and reporting data. In fact, there should be more of an expectation that data from all treatments, not just overtly experimental ones, is routinely collected and made available for research. It has been suggested that, given the issues posed by emerging modes of research and translation, we need to reconceptualize research participation to emphasize social utility as well as individual choice: especially when it comes to the use of health data, which poses much lower physical risks, there ought to be a presumption in favor of research and of using all relevant information [72].

## Selling science

Another area that often raises warning flags with respect to stem cell treatments is pay-to-participate studies. Here, we must ask: is placing (part of) the economic costs of research onto participants inherently unethical? Although previous analyses have noted possible concerns [73,74], it seems that pay-to-participate research is not necessarily ethically unacceptable [75,76]. Even so, however, we need to consider how and under what circumstances it should be acceptable for participants to pay for inclusion, and whether the current provision of pay-to-participate stem cell treatments meets these criteria.

There are questions of justice here that cut both ways: it might be considered unfair to the participants who are included, in the sense that the costs as well as the risks of research are borne by those already unfortunate enough to suffer grave disease. On the other hand, making patients’ eligibility to take part in trials dependent on their ability to pay affects who is able to participate; if research participation is seen as a good in itself [72,77,78], this has implications for equitable access, creating unfairness for those who are excluded.

Another potential objection to pay-to-participate is that the introduction of a commercial element to the process distorts participants’ perceptions of what is happening. It is known that paying for something changes perceptions of value; in this case, paying to receive an experimental treatment may give patients mistaken ideas about the treatment's effectiveness. This has implications for the scientific validity of any research carried out, as well as ethical consequences in terms of therapeutic misconception [74].

We might also say that allowing pay-to-participate studies misleads patients because it gives them the illusion of control, over their condition and over the possible avenues of treatment open to them. Here, however, we must consider what control means for patients in often desperate situations. Granted, research participation does not give patients control in terms of assuring an effective treatment, but a sense of control itself is something experiential, subjective rather than objective. The idea of being able to do something, whether or not that something is effective, may be an important way for patients to exercise some agency in a situation where there is very little else they can do [29].

Once again, however, we need to be wary of false advertising in relation to what is being offered as research for participation. If patients are given the impression that they are taking part in research on a pay-to-participate basis but there is no real science going on, what they are being sold is not participation.

The idea of paying to receive treatment as part of a study raises a broader issue of treating health as a consumer product. While improving equity of access to health is seen as a fundamental ethical goal, we accept more unevenness in the consumer market, perhaps because it deals with less essential needs, goods for which unequal distribution is not so important. A question raised by pay-to-participate schemes is thus whether access to science and research participation should be a consumer product.

Based on ideas about the importance of science as a social process, we might argue that participation in science ought to be perceived as a basic good more akin to health, to which all should ideally have equitable access, rather than a consumer good available based on ability to pay [72,79]. This does not exclude any possibility of participant-funded research, but does mean we should consider carefully whether pay-to-participate schemes are jeopardizing access to science in an unjust way.

There are thus many reasons to be critical and cautious about the idea of pay-to-participate schemes for stem cell therapies and regenerative medicine. Nevertheless, condemning such activities may not have much effect on patients’ actions. They are not the ones who determine whether or not they are charged for a treatment; all that is open to them to decide is whether, when the treatments are being provided on a charged basis, they will pay to receive them. From the patients’ perspective, the choice is not whether they should pay or not pay to receive an experimental treatment; it is whether they pay and receive treatment versus nothing; and when the alternative is nothing, it seems that at least some are electing to pay.

## Conclusion

The currents emerging in regenerative medicine across all the areas discussed show that there is a clear desire on the part of patients to be more active agents not only in managing their own treatments but also the process of research and developing new treatments. The question, then, is what is needed to support this and direct it into productive channels that will help to attain the eventual goal of successful regenerative medicine treatments? As evidence demonstrates, focusing on blocking channels that are seen as unproductive is more likely simply to divert this force elsewhere.

Regarding the role of patients in healthcare and health research, the present dilemmas faced in regenerative medicine, among other areas of science, signal the need for an ethical reconceptualization of research and our rights and responsibilities with respect to science, both as patients and as participants. In relation to stem cell therapies, an examination of the role of patients as health consumers, how the triple role of patient–participant–consumer is negotiated in transactions and interactions with others and how this impacts the direction of the field is also needed.

Likewise, understanding the dynamics of regenerative medicine will require exploration of the parallel set of relationships between research, healthcare provision and the market and how these influence the processes of discovery, translation and innovation. This may help to address problems such as how to best meet the costs and risks of scaling up, from small clinical studies with individual patients, to the point where standardized treatments are widely available.

Finally, consideration must be given to the role of regulation in shaping stem cell innovation. Although transnational regulatory differences continue to have an effect on the regenerative medicine field, harmonization is not necessarily the best way forward. We must also consider in which areas regulation can best be targeted – healthcare, medical research, the consumer market – and how regulation across these different areas can be engineered to work smoothly in concert. Lastly, there is also the question of what level of regulation is required and what measures will be most effective in achieving the desired changes in practice. From high-level law, through regulatory processes of bodies such as the FDA and the UK's NICE, and guidelines issued by professional organizations, to non-regulatory interventions that bring about changes in behavior and daily practice, it may be that it is not regulation, or at least not regulation alone, that can have most impact in this area.

## Future perspective

The next few years are likely to see further shifts in the relationships between publics, patients, biomedicine and healthcare, particularly at the research translation interface. Commercial and regulatory factors will continue to influence these interactions. With respect to regenerative medicine, the aim should be to achieve mutual cooperation between patients and researchers in order to work toward the common goal of effective therapies. Ethics and social science can assist in this process by enabling us to understand the complex dynamics at play, in order to promote constructive engagement.

**Executive summary**

**Background/defining the problem**

- The provision of unproven cell therapies continues to be a major ethical challenge in the field of regenerative medicine.

**From stem cell tourism to trouble at home**

- New tensions are becoming apparent between different stakeholder groups, particularly patients who are seeking to receive treatments and scientists who advocate tighter restrictions on provision.

**Tapping the multinational research market**

- Transnational differences in the regulation of regenerative medicine reflect national strategies for scientific development in the context of global competition, but may enable research and innovation tourism that can have adverse effects on global scientific equity.

**Patient rights & access to treatment: a new role as health consumers**

- Patient rights are assuming a larger role in the discourse, with the emergence of the health consumer role signaling a shift in the terms on which publics are seeking to access treatments.

**Translational strategies for regenerative medicine?**

- If regenerative medicine is to succeed, it may require the exploration of new translational pathways, given the combination of scientific, social, political and economic factors at play.
- Reframing the relationships between publics and sciences and their roles with respect to research, innovation and the governance of science may help to navigate the challenges ahead.
